# Divergent Clonal Trajectories of *FLT3*‐ITD in Acute Myeloid Leukemia and Their Clinical and Transcriptomic Associations

**DOI:** 10.1002/hon.70261

**Published:** 2026-09-22

**Authors:** Xavier Cheng‐Hong Tsai, Yu‐Sung Chang, Feng‐Ming Tien, Min‐Yen Lo, Yuan‐Yeh Kuo, Mei‐Hsuan Tseng, Yen‐Ling Peng, Chi‐Yuan Yao, Szu‐Chi Yao, Chien‐Chin Lin, Bor‐Sheng Ko, Ming Yao, Hwei‐Fang Tien, Hsin‐An Hou, Wen‐Chien Chou

**Affiliations:** ^1^ Division of Hematology Department of Internal Medicine National Taiwan University Hospital Taipei Taiwan; ^2^ Department of Hematology Oncology National Taiwan University Cancer Center, National Taiwan University Hospital Taipei Taiwan; ^3^ Graduate Institute of Clinical Medicine College of Medicine National Taiwan University Taipei Taiwan; ^4^ Division of Hematology Department of Internal Medicine National Taiwan University Hospital Yunlin Branch Yunlin Taiwan; ^5^ Tai‐Chen Cell Therapy Center National Taiwan University Taipei Taiwan; ^6^ Division of Hematological Oncology Department of Internal Medicine Chi‐Mei Medical Center Tainan Taiwan; ^7^ Department of Laboratory Medicine National Taiwan University Hospital Taipei Taiwan; ^8^ Department of Internal Medicine Far‐Eastern Memorial Hospital New Taipei City Taiwan; ^9^ Division of General Medicine Department of Internal Medicine National Taiwan University Hospital Taipei Taiwan

**Keywords:** acute myeloid leukemia, clonal evolution, *FLT3*mutation, prognostication, transcriptomics

## Abstract

*FLT3*‐ITD occurs in approximately 20%–25% of cases of adult AML and is associated with increased relapse risk and shorter survival. *FLT3*‐ITD status informs AML risk at diagnosis, but its dynamic changes during disease evolution are not well defined. We hypothesized that divergent *FLT3*‐ITD clonal trajectories are shaped by preexisting molecular features detectable at diagnosis. We analyzed 319 intensively treated adults who were newly diagnosed with non‐M3 *de novo* AML who experienced relapse and had paired *FLT3*‐ITD mutation data at diagnosis and relapse. Patients were classified into four clonal evolution patterns: maintained *FLT3*‐ITD negative (*FLT3*‐ITD^negative^), *FLT3*‐ITD loss (*FLT3*‐ITD^lost^), *FLT3*‐ITD acquisition (*FLT3*‐ITD^acquired^), and maintained *FLT3*‐ITD positive (*FLT3*‐ITD^positive^). The *FLT3*‐ITD^positive^ group was strongly associated with concurrent *NPM1* and/or *DNMT3A* mutations, which were enriched compared with those in other groups (*p* < 0.001). ELN 2022 risk categories differed across clonal evolution patterns, with favorable‐risk disease declining progressively from the *FLT3*‐ITD^negative^ to the *FLT3*‐ITD^positive^ group (44.3%–6.1%) while intermediate‐risk disease rose correspondingly (21.2%–73.5%; both *p* < 0.001). With a median follow‐up of 96.8 months, patients in the *FLT3*‐ITD^acquired^ or *FLT3*‐ITD^positive^ group had significantly inferior relapse‐free and overall survival compared with those in the *FLT3*‐ITD^negative^ group (*p =* 0.009 and *p =* 0.018, respectively, in univariate analyses; *p =* 0.001 and *p =* 0.006, respectively, in multivariate analyses adjusted for individual genetic risk features). Transcriptomics showed enrichment of inflammatory pathways in *FLT3*‐ITD^positive^ cases that persisted after adjustment for *NPM1*/*DNMT3A* co‐mutations and *FLT3*‐ITD allelic ratio, together with an inferred monocyte‐rich profile. Overall, *FLT3*‐ITD evolution is associated with distinct genomic and transcriptomic features present at diagnosis.

**Trial Registration:**

202203013RSD

## 1 Introduction

Acute myeloid leukemia (AML) is a biologically and clinically heterogeneous and aggressive hematologic malignancy [1] characterized by the abnormal proliferation and differentiation of progenitor cells. Among the myriad genetic alterations observed in patients with AML, mutations in *FMS‐like tyrosine kinase 3* (*FLT3*), particularly internal tandem duplications (ITDs), are of paramount clinical importance because of their association with adverse prognosis [2, 3, 4, 5]. Despite intensive chemotherapy and allogeneic hematopoietic stem cell transplantation (HSCT), patients with *FLT3*‐ITD consistently have poor outcomes [4, 6]. In recent years, the incorporation of *FLT3* inhibitors into both intensive and lower‐intensity treatment strategies has substantially improved response rates and survival [7, 8, 9]; however, durable long‐term remission remains a major clinical challenge.

However, *FLT3*‐ITD status is not static. AML often harbors multiple subclones, and under the selective pressure of cytotoxic therapy or targeted therapy, the clonal architecture evolves dynamically [10, 11, 12]. Evidence suggests that *FLT3*‐ITD can be lost or acquired at relapse, and the reported rates vary significantly across studies [10, 12, 13]. While the emergence of resistance mutations under treatment with *FLT3* inhibitors is well documented [11, 14], the patterns of clonal evolution under standard intensive chemotherapy remain less comprehensively characterized, particularly in large, real‐world cohorts. Understanding these dynamic shifts, specifically differentiating between patients who lose the mutation and patients who acquire it, is crucial for determining prognosis and guiding salvage therapy, as these differences likely reflect distinct mechanisms of therapeutic resistance. The loss of *FLT3*‐ITD at relapse, observed in a subset of patients, often signifies the elimination of the sensitive *FLT3*‐mutated clone and the subsequent selection of a chemoresistant wild‐type subclone, potentially driven by alternative pathways such as the *RAS/MAPK* signaling axis [10, 11, 14]. In contrast, the acquisition of *FLT3*‐ITD or the expansion of *FLT3*‐ITD microclones in patients initially diagnosed with the wild type typically represents the expansion of a minor, previously undetectable subclone that gains a fitness advantage under therapeutic pressure [15].

We hypothesized that these trajectories are not stochastic but are shaped by preexisting molecular and transcriptomic programs that are detectable at the time of diagnosis. Such predictive capabilities could stratify high‐risk patients earlier and optimize monitoring strategies. In this study, we provide a comprehensive analysis of *FLT3*‐ITD clonal evolution in a cohort of AML patients who were treated with intensive chemotherapy, with serial samples collected during follow‐up. We categorized patients into distinct evolutionary patterns (maintained negative, loss, acquisition, and maintained positive, denoted as *FLT3*‐ITD^negative^, *FLT3*‐ITD^lost^, *FLT3*‐ITD^acquired^, *FLT3*‐ITD^positive^, respectively) to evaluate the specific effects of these patterns on clinical outcomes. We further investigated diagnostic transcriptomic profiles to elucidate biological pathways and microenvironmental differences that potentially drive the clonal evolution of *FLT3*‐ITD–positive clones.

## 2 Methods

### 2.1 Study Design

This multicenter, retrospective, observational cohort study included adult patients with non‐M3 *de novo* acute myeloid leukemia (AML) treated at the National Taiwan University Hospital (NTUH), including its Yunlin Branch and the National Taiwan University Cancer Center, between 1995 and 2020. Only patients whose diagnosis, treatment, and subsequent relapse were managed at NTUH or its affiliated branches were included. The diagnosis was confirmed according to the World Health Organization (WHO) 2022 classification [16] and International Consensus Classification (ICC) [17]. Relapse was defined as bone marrow blasts ≥ 5% after remission was achieved [18]. Patients were excluded if they had a history of myelodysplastic syndromes/neoplasms or myeloproliferative neoplasms, had no record of *FLT3* testing, had secondary or therapy‐related AML, or less than 5% blasts in bone marrow at relapse.

For frontline therapy, patients who were deemed fit for intensive chemotherapy were subjected to a 3 + 7 induction regimen, with the option of a less intensive 2 + 5 regimen for older patients. Owing to reimbursement constraints during the study period, patients did not receive *FLT3* inhibitors as part of frontline treatment. Postremission therapy included 2–4 courses of high‐dose cytarabine, administered with or without anthracycline. Only patients who received intensive chemotherapy were included in the survival analysis. Treatment responses were evaluated according to European LeukemiaNet (ELN) recommendations [18]. The choice to proceed with allogeneic hematopoietic stem cell transplantation was dictated by molecular risk stratification, age, comorbidities, donor availability, and patient response to induction treatment, which was assessed through morphological observation and multicolor flow cytometry, a standard procedure at our institute. Post‐transplant maintenance therapy with *FLT3* inhibitors was likewise unavailable during the study period.

### 2.2 Molecular and Bioinformatic Analyses

Next‐generation sequencing (NGS) was conducted with a TruSight myeloid sequencing panel and HiSeq platform (Illumina, San Diego, CA). Library preparation and sequencing followed the manufacturer's protocols, achieving a median reading depth of 11,000× [19, 20]. The variant analysis algorithm for diagnostic samples, which was previously detailed [19, 21], used a minimum variant allele frequency of 5%. *FLT3*‐ITD status was assessed at initial diagnosis with a sensitivity of 1% [22] and was re‐evaluated at relapse. In patients with archived diagnostic material, ITD lesions were additionally characterized by amplicon‐based deep sequencing with getITD [23]. A total of 132 patients had cryopreserved diagnostic bone marrow samples available for RNA sequencing analysis (Supporting Information S1: Table S1). Libraries were prepared using the TruSeq Stranded mRNA Library Prep Kit (Illumina, San Diego, CA) and sequenced on an Illumina NovaSeq 6000 platform. To investigate the baseline transcriptomic differences between patients who lost *FLT3*‐ITD and those who maintained it at relapse, bulk RNA‐sequencing data were analyzed. Differential expression analysis was performed using DESeq2, with significance defined as an absolute fold change > 2 and an adjusted *p* value < 0.01; the same adjusted *p* threshold was applied to pathway‐level results. To address potential confounding by co‐mutation status and clone burden, the analysis was repeated with *NPM1*/*DNMT3A* mutation status, and *FLT3*‐ITD allelic ratio (AR) included as covariates in the design formula, both individually and jointly. Gene set entrichment analysis (GSEA) was repeated on the ranked test statistics derived from each adjusted model. For the four‐group comparison, a joint DESeq2 model was fitted, and a likelihood ratio test (LRT) against the intercept‐only reduced model was used. Pairwise contrasts were extracted from the same model, and GSEA was performed on genes ranked by the Wald statistic from the LRT‐fitted model. Single‐sample enrichment scores were computed using GSVA and correlated with continuous *FLT3*‐ITD AR by Spearman correlation. The transcriptomes were further analyzed using CIBERSORTx [24] to infer immune cell type composition by digital cytometry. Other detailed information is provided in the Supporting Information S1: Methods.

### 2.3 Statistical Analyses

Continuous variables and medians were compared using the Mann‒Whitney *U* test or the Kruskal‒Wallis test. Differences between discrete variables were analyzed with the chi‐square test or Fisher's exact test. Overall survival (OS) was defined from the date of initial diagnosis to the date of last follow‐up or death from any cause, whereas relapse‐free survival (RFS) was defined from the date of initial diagnosis to the date of hematologic relapse or death from any cause, whichever occurred first. Survival probabilities were calculated using Kaplan‒Meier analysis, and the log‐rank test was used to assess statistical significance. The Cox proportional hazards model was employed for multivariate regression analysis and generated hazard ratios (HRs) and 95% confidence intervals (CIs). All the statistical analyses were conducted using R version 4.4.0 (https://cran.r‐project.org/). A two‐sided *p* value of less than 0.05 was considered to indicate statistical significance.

## 3 Results

Among the 1733 patients with AML who were screened, 319 experienced relapse and had available *FLT3* mutation data at both diagnosis and relapse (Supporting Information S1: Figure S1). The clinical characteristics were analyzed on the basis of the evolution of the *FLT3*‐ITD status and were categorized into four groups: *FLT3*‐ITD^negative^ (*n* = 212, 66.5%), *FLT3*‐ITD^lost^ (*n* = 27, 8.5%), *FLT3*‐ITD^acquired^ (*n* = 31, 9.7%), and *FLT3*‐ITD^positive^ (*n* = 49, 15.4%). A detailed comparison of these groups, including their clinical features and prognostic implications, is presented in Table 1 and Supporting Information S1: Table S2.

**TABLE 1:** Comparison of clinical characteristics of relapsed AML patients with different *FLT3*‐ITD evolution patterns.

Variables	*FLT3*‐ITD^negative^ (*n* = 212, 66.5%)	*FLT3*‐ITD^acquired^ (*n* = 31, 9.7%)	*FLT3*‐ITD^lost^ (*n* = 27, 8.5%)	*FLT3*‐ITD^positive^ (*n* = 49, 15.4%)	*p* value
Sex^a^					> 0.999
Male	110 (51.9%)	16 (51.6%)	14 (51.9%)	26 (53.1%)	
Female	102 (48.1%)	15 (48.4%)	13 (48.1%)	23 (46.9%)	
Age (year)^b^	48.5 (16.1–84.0)	38.2 (18.4–70.4)	45.5 (17.8–68.6)	51.8 (19.3–85.9)	0.160
Lab^b^					
WBC (/μL)	13,935 (490–361,300)	13,530 (760–266,130)	26,050 (810–206,970)	50,560 (1670–377,130)	< 0.001
Hb (g/dL)	8.35 (2.7–13.5)	8.0 (5.8–14.2)	7.3 (4.6–12.1)	8.0 (3.6–11.5)	0.169
Platelet (× 1000/μL)	46 (3–712)	39 (8–366)	41 (7–128)	58 (5–436)	0.410
Peripheral blood blast (/μL)	6314 (0–352,268)	3316 (0–212,904)	20,840 (66–162,629)	33,570 (0–358,651)	< 0.001
LDH (U/L)	695 (96–7747)	750 (145–4952)	564 (167–3353)	785 (289–7177)	0.072
*FLT3*‐ITD allelic ratio median			0.21 (0.01–10.60)	0.76 (0.01–22.77)	< 0.001
High *FLT3*‐ITD allelic ratio^c^			9 (33.3%)	36 (73.5%)	0.001
FAB^a^					
M0	4 (1.9%)	0 (0%)	1 (3.7%)	0 (0%)	0.529
M1	51 (24.1%)	5 (16.1%)	7 (25.9%)	11 (22.4%)	0.781
M2	88 (41.5%)	12 (38.7%)	10 (37.0%)	14 (28.6%)	0.416
M4	58 (27.4%)	9 (29.0%)	8 (29.6%)	19 (38.8%)	0.475
M5	8 (3.8%)	1 (3.2%)	0 (0%)	5 (10.2%)	0.140
M6	3 (1.4%)	4 (12.9%)	1 (3.7%)	0 (0%)	0.007
2017 ELN^a^					
Favorable	93 (43.9%)	11 (35.5%)	7 (25.9%)	11 (22.4%)	0.020
Intermediate	61 (28.8%)	13 (41.9%)	10 (37.0%)	24 (49.0%)	0.037
Adverse	58 (27.4%)	7 (22.6%)	10 (37.0%)	14 (28.6%)	0.660
2022 ELN^a^					
Favorable	94 (44.3%)	11 (35.5%)	4 (14.8%)	3 (6.1%)	< 0.001
Intermediate	45 (21.2%)	12 (38.7%)	16 (59.3%)	36 (73.5%)	< 0.001
Adverse	73 (34.4%)	8 (25.8%)	7 (25.9%)	10 (20.4%)	0.215
HSCT at CR1^a^	28 (13.2%)	4 (12.9%)	10 (37.0%)	7 (14.3%)	0.028
NGS MRD available patient number	85	15	11	16	
NGS MRD negativity after induction	32 (37.6%)	7 (46.7%)	8 (72.7%)	9 (56.2%)	0.107
NGS MRD negativity after 1st consolidation	52 (61.2%)	9 (60.0%)	9 (81.8%)	9 (56.2%)	0.550

Abbreviations: AML, acute myeloid leukemia; CR, complete remission; ELN, European LeukemiaNet; FAB, French‐American‐British classification; Hb, hemoglobin; LDH, lactate dehydrogenase; MRD, measurable/minimal residual disease; WBC, white blood cell.

^a^ number of patients (%).

^b^ median (range).

^c^ *FLT3*‐ITD allelic ratio > 0.5 was categorized as high allelic ratio.

The WBC count was greater in the *FLT3*‐ITD^positive^ group than in the *FLT3*‐ITD^negative^ group (*p* < 0.001). Similarly, peripheral blood blast counts were significantly greater in the *FLT3*‐ITD^positive^ group than in the other groups (*p* < 0.001). Compared with the *FLT3*‐ITD^lost^ group, patients in the *FLT3*‐ITD^positive^ group had a higher *FLT3*‐ITD burden (*p* < 0.001) and a greater proportion of cases with a high AR (*p =* 0.001). Restricting the analysis to the 76 patients who were *FLT3*‐ITD–positive at diagnosis, AR did not predict maintained positivity versus loss either alone (*p =* 0.307) or alongside co‐mutation status (*p =* 0.827), whereas concurrent *NPM1* and *DNMT3A* mutations did (*p =* 0.014). The FAB classification varied: the M4 subtype was more prevalent in the *FLT3*‐ITD^positive^ group (38.8%) than in the other groups. Genetic analysis revealed that the *FLT3*‐ITD^positive^ group had a notably higher frequency of *NPM1* mutations (61.2%) than the other groups, in which the frequencies ranged from 13.7% to 25.9% (*p* < 0.001). Moreover, compared with the other groups, the *FLT3*‐ITD^negative^ group had a higher frequency of AML with in‐frame bZIP *CEBPA* mutations (*p* = 0.049). Interestingly, concurrent *NPM1* and *DNMT3A* mutations were most frequently observed in the *FLT3*‐ITD^positive^ group (40.8%) compared with the *FLT3*‐ITD^negative^ (6.6%), *FLT3*‐ITD^acquired^ (6.5%), and *FLT3*‐ITD^lost^ groups (7.4%) (*p* < 0.001). Per the 2022 ELN risk classification, favorable‐risk disease declined progressively from the *FLT3*‐ITD^negative^ (44.3%) through the *FLT3*‐ITD^acquired^ (35.5%) and *FLT3*‐ITD^lost^ (14.8%) to the *FLT3*‐ITD^positive^ group (6.1%; *p* < 0.001), with a reciprocal increase in intermediate‐risk disease (21.2%, 38.7%, 59.3%, and 73.5%, respectively; *p* < 0.001). The proportion of adverse‐risk disease did not differ significantly across groups. Because no patient in this cohort received a *FLT3* inhibitor, we additionally classified the cohort according to the 2017 ELN criteria. The same directional pattern was present but was considerably attenuated (Table 1). The proportion of patients who underwent HSCT in the first complete remission (CR1) was significantly greater in the *FLT3*‐ITD^lost^ group (37.0%) than in the other groups (*p* = 0.028). Given that the cohort consisted exclusively of patients who relapsed, *FLT3*‐ITD loss was observed through two clinical pathways: approximately half of the patients harbored *FLT3*‐ITD at diagnosis, underwent HSCT in CR1, and subsequently relapsed with loss of the mutation; the remaining half also presented with *FLT3*‐ITD at diagnosis, experienced relapse characterized by loss of the mutation, and then underwent HSCT. To investigate whether prior HSCT was associated with *FLT3*‐ITD clonal eradication, we compared post‐transplant versus non‐transplant relapses within patients with *FLT3*‐ITD at diagnosis (*n* = 76). Post‐transplant relapses were significantly more likely to be *FLT3*‐ITD negative (10/17, 58.8%) than non‐transplant relapses (17/59, 28.8%) (*p =* 0.042) (Supporting Information S1: Table S3).

**FIGURE 1:**
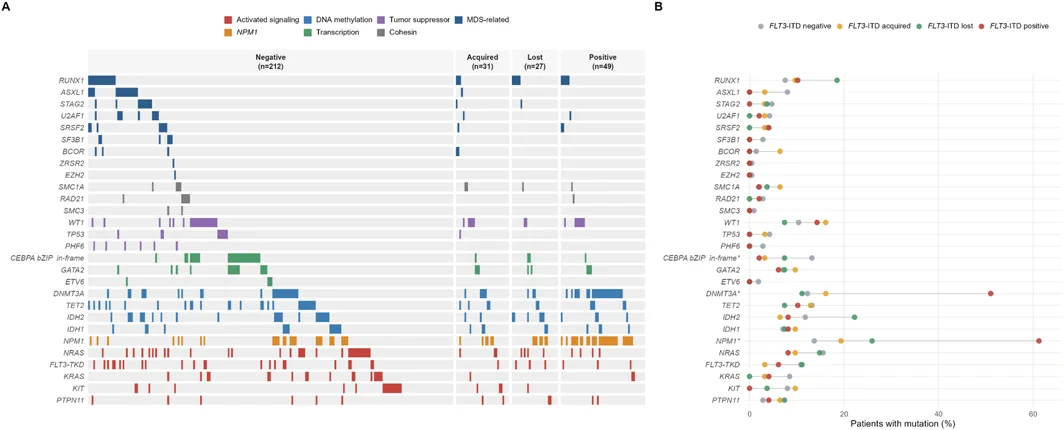
(A) Oncoprint representation of the genes assessed at diagnosis. Each column represents one patient and each row one gene. (B) Frequency of each mutation within each trajectory group. Each gene is represented by four points, one per group, on a common axis. Asterisks denote genes whose frequency differed significantly across the four trajectories.

Across the *FLT3*‐ITD trajectory groups, patients within *FLT3*‐ITD^negative^, *FLT3*‐ITD^acquired^, or *FLT3*‐ITD^lost^ groups showed heterogeneous mutational profiles without clear enrichment of specific cooperating mutations. In contrast, *FLT3*‐ITD^positive^ patients had substantially higher rates of *NPM1* and *DNMT3A* mutations (both *p* < 0.001; Figure 1 and Table 2). The total co‐mutation count did not differ significantly across trajectory groups (*p =* 0.238). Interestingly, when *NPM1* and *DNMT3A* were excluded from the count, the *FLT3*‐ITD^positive^ group carried the fewest additional co‐mutations (median 1, interquartile range (IQR) 0–1), compared with median 1 (IQR 1–2) in each of the other three groups (*p =* 0.001). To characterize clonal co‐mutation dynamics at the patient level, we performed paired mutation analysis for 29 *FLT3*‐ITD^acquired^ and 21 *FLT3*‐ITD^lost^ patients. No co‐mutation was detected at either time point in 16 (55%) and 10 (48%) respectively. Among the 45 co‐mutations present at diagnosis in the remaining patients, 27 (60%) were retained at relapse and 5 further mutations emerged. Mutations in DNA methylation genes were almost always retained (10 of 11, 91%), whereas activated signaling mutations were usually lost (3 of 10, 30%; *p =* 0.008), and all 5 signaling mutations present in the *FLT3*‐ITD^lost^ group disappeared together with the ITD clone (Supporting Information S1: Table S4).

**TABLE 2:** Comparison of gene mutations between relapsed AML patients with different *FLT3*‐ITD evolution patterns.

Categories	Genes	*FLT3*‐ITD^negative^ (*n* = 212, 66.5%)	*FLT3*‐ITD^acquired^ (*n* = 31, 9.7%)	*FLT3*‐ITD^lost^ (*n* = 27, 8.5%)	*FLT3*‐ITD^positive^ (*n* = 49, 15.4%)	*p* value
Activated signaling	*FLT3*‐TKD	23 (10.8%)	1 (3.2%)	3 (11.1%)	3 (6.1%)	0.524
	*KIT*	17 (8.0%)	3 (9.7%)	1 (3.7%)	0 (0.0%)	0.108
	*NRAS*	33 (15.6%)	3 (9.7%)	4 (14.8%)	4 (8.2%)	0.566
	*KRAS*	18 (8.5%)	1 (3.2%)	0 (0%)	2 (4.1%)	0.345
	*PTPN11*	6 (2.8%)	2 (6.5%)	2 (7.4%)	2 (4.1%)	0.326
Tumor suppressor	*WT1*	22 (10.4%)	5 (16.1%)	2 (7.4%)	7 (14.3%)	0.619
	*TP53*	9 (4.2%)	1 (3.2%)	0 (0%)	0 (0%)	0.452
	*PHF6*	6 (2.8%)	0 (0%)	0 (0%)	0 (0%)	0.792
	*NPM1*	29 (13.7%)	6 (19.4%)	7 (25.9%)	30 (61.2%)	< 0.001
DNA methylation	*IDH1*	15 (7.1%)	3 (9.7%)	2 (7.4%)	4 (8.2%)	0.914
	*IDH2*	25 (11.8%)	2 (6.5%)	6 (22.2%)	4 (8.2%)	0.268
	*DNMT3A*	26 (12.3%)	5 (16.1%)	3 (11.1%)	25 (51.0%)	< 0.001
	*TET2*	28 (13.2%)	4 (12.9%)	2 (7.4%)	5 (10.2%)	0.886
Cohesin	*RAD21*	6 (2.8%)	0 (0%)	0 (0%)	1 (2.0%)	> 0.999
	*SMC1A*	4 (1.9%)	2 (6.5%)	1 (3.7%)	1 (2.0%)	0.288
	*SMC3*	2 (0.9%)	0 (0%)	0 (%)	0 (0%)	> 0.999
Transcription	*GATA2*	15 (7.1%)	3 (9.7%)	2 (7.4%)	3 (6.1%)	0.925
	*CEBPA* bZIP in‐frame	28 (13.2%)	1 (3.2%)	2 (7.4%)	1 (2.0%)	0.049
	*ETV6*	4 (1.9%)	0 (0%)	0 (0%)	0 (0%)	> 0.999
MDS‐related mutations	*ASXL1*	17 (8.0%)	1 (3.2%)	0 (0%)	0 (%)	0.073
	*BCOR*	3 (1.4%)	2 (6.5%)	0 (0%)	0 (0%)	0.194
	*EZH2*	1 (0.5%)	0 (0%)	0 (0%)	0 (0%)	> 0.999
	*RUNX1*	16 (7.5%)	3 (9.7%)	5 (18.5%)	5 (10.2%)	0.288
	*SF3B1*	6 (2.8%)	0 (0%)	0 (0%)	0 (0%)	0.792
	*SRSF2*	8 (3.8%)	1 (3.2%)	0 (0%)	2 (4.1%)	0.904
	*STAG2*	10 (4.7%)	1 (3.2%)	1 (3.7%)	0 (0%)	0.440
	*U2AF1*	9 (4.2%)	1 (3.2%)	0 (0%)	1 (2.0%)	0.860
	*ZRSR2*	1 (0.5%)	0 (0%)	0 (0%)	0 (0%)	> 0.999

Amplicon‐based deep sequencing with dedicated ITD calling was performed on archived material from 46 *FLT3‐*ITD^positive^ and 25 *FLT3*‐ITD^lost^ patients. Most patients carried more than one ITD lesion at diagnosis, and neither the number of lesions, the ITD length, nor the insertion site differed between the two trajectories (Supporting Information S1: Table S5). Among 42 patients sequenced at both time points, the dominant diagnostic lesion remained detectable at relapse in 37 (88.1%); in 3 the relapse population derived from a minor diagnostic subclone, and in 2 from an ITD not detectable at diagnosis. Considering the 118 individual ITDs detected at diagnosis, abundance was the only determinant of persistence (odds ratio 5.00 per ten‐fold increase in variant allele frequency, *p* < 0.001); neither ITD length nor insertion site was associated with survival to relapse.

**FIGURE 2:**
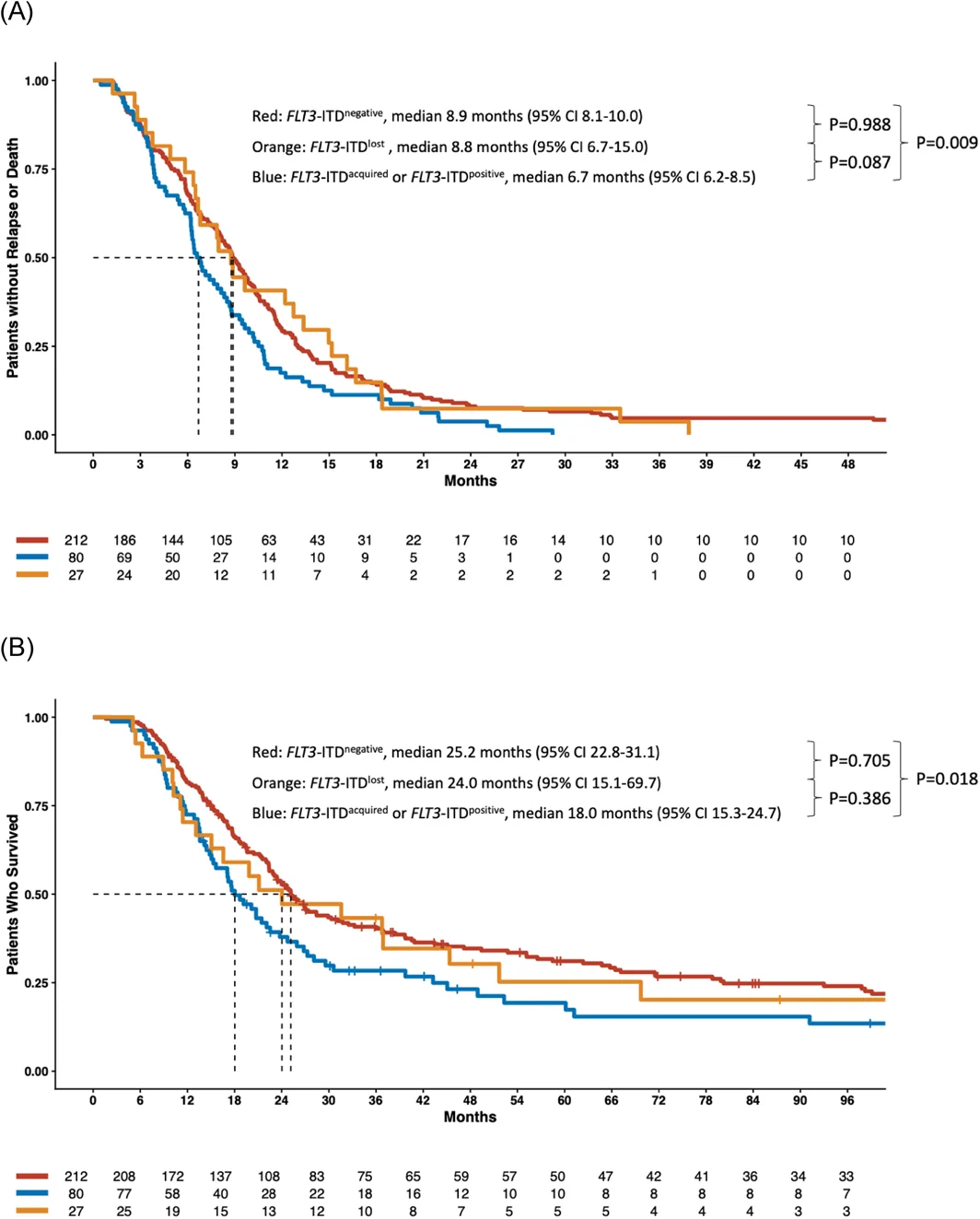
(A) Relapse‐free survival and (B) overall survival stratified by *FLT3*‐ITD clonal evolution patterns.

We further analyzed OS stratified by clonal evolution patterns. With a median follow‐up of 96.8 (range 0.1–260.67) months, we found that *FLT3*‐ITD^acquired^ or *FLT3*‐ITD^positive^ patients had significantly shorter RFS and OS than *FLT3*‐ITD^negative^ patients did (*p* = 0.009 and 0.018, respectively; Figure 2). In multivariate Cox proportional hazards regression analysis, we included age, genetic risk features, *FLT3*‐ITD clonal evolution, and HSCT at CR1 as covariates (Table 3). Because the 2022 ELN risk classification incorporates *FLT3*‐ITD status, adjusting for the composite ELN category when *FLT3*‐ITD trajectory is the exposure of interest would constitute overadjustment. We therefore adjusted for the individual molecular alterations, including cytogenetic changes, in‐frame bZIP *CEBPA*, *NPM1*, *TP53*, and myelodysplasia‐related gene mutations. In this model, *FLT3*‐ITD^acquired^ or *FLT3*‐ITD^positive^ remained an independent adverse prognosticator for both RFS and OS (*p =* 0.001 and *p =* 0.006, respectively, Table 3). The finding was consistent when the ELN 2022 framework was retained but its *FLT3*‐ITD criteria were removed (RFS, *p =* 0.003; OS, *p =* 0.012; Supporting Information S1: Table S6). Similar finding was demonstrated in terms of ELN 2017 risk classification (RFS, *p =* 0.011 and OS, *p =* 0.022, respectively; Supporting Information S1: Table S7). Within the 76 patients who were *FLT3*‐ITD–positive at diagnosis, a higher AR was independently associated with shorter RFS and OS (HR = 1.084 per 0.1 increase, *p =* 0.011 and *p =* 0.008; Supporting Information S1: Table S8).

**TABLE 3:** Multivariate cox proportional hazards regression analyses.

Variables	Relapse‐free survival				Overall survival			
	HR	Lower	Upper	*p* value	HR	Lower	Upper	*p* value
Age^a^	1.007	0.999	1.014	0.081	1.017	1.008	1.026	< 0.001
Cytogenetic risk group^b^								
Favorable	Reference				Reference			
Intermediate	0.998	0.703	1.417	0.992	1.298	0.866	1.948	0.207
Adverse	1.317	0.873	1.986	0.190	1.550	0.964	2.494	0.071
Gene mutations^c^								
In‐frame bZIP *CEBPA*	0.992	0.659	1.493	0.968	0.621	0.379	1.018	0.059
Mutated *NPM1*	0.844	0.619	1.150	0.282	0.612	0.423	0.886	0.009
Mutated *TP53*	2.629	1.330	5.197	0.005	3.836	1.903	7.730	< 0.001
Myelodysplasia‐related gene mutation	1.112	0.833	1.483	0.471	1.000	0.720	1.389	> 0.999
*FLT3*‐ITD clonal evolution								
*FLT3*‐ITD^lost^ versus *FLT3*‐ITD^negative^	1.209	0.791	1.847	0.381	1.058	0.640	1.751	0.825
*FLT3*‐ITD^acquired^ or *FLT3*‐ITD^positive^ versus *FLT3*‐ITD^negative^	1.561	1.186	2.055	0.001	1.551	1.136	2.119	0.006
HSCT at CR1^d^	1.138	0.816	1.585	0.447	1.601	1.103	2.323	0.013

Abbreviations: CI, confidence interval; CR, complete remission; ELN, European LeukemiaNet; HR, hazard ratio; HSCT, hematopoietic stem cell transplantation.

^a^ Continuous variable.

^b^ According to the refined Medical Research Council (MRC) criteria.

^c^ mutated versus wild‐type.

^d^ HSCT at CR1 versus HSCT at other disease status or without HSCT, modeled as a time‐dependent covariate.

To determine whether the baseline transcriptomic landscape was associated with subsequent *FLT3*‐ITD clonal evolution, we compared diagnostic RNA‐seq profiles between patients who lost versus maintained *FLT3*‐ITD at relapse. Hierarchical clustering revealed distinct expression patterns between the two groups (Figure 3A). Differential expression analysis highlighted several significantly dysregulated genes, including *EPHB2*, *MECOM*, *POU4F1*, *LGALS3*, *FN1*, and *KLF4* (fold change > 2, adjusted *p* < 0.01; Figure 3B). Given the enrichment of *NPM1* and *DNMT3A* co‐mutations and differences in *FLT3*‐ITD AR between groups, we repeated the analysis with these variables as covariates (Supporting Information S1: Table S9 and Figure S2). After adjustment, *MECOM* was the only gene retaining significance (adjusted *p =* 1.8 × 10^−4^). Consistently, in an expanded analysis across all four trajectory groups, *MECOM* was also significantly downregulated in *FLT3*‐ITD^positive^ versus *FLT3*‐ITD^negative^ patients (adjusted *p =* 1.4 × 10^−11^).

FIGURE 3Unadjusted transcriptomic and immune microenvironment analysis of diagnostic samples from patients in the *FLT3*‐ITD^positive^ and *FLT3*‐ITD^lost^ groups. (A) Hierarchical clustering and heatmap of differentially expressed genes between patients in the *FLT3*‐ITD^lost^ group (blue) and those in the *FLT3*‐ITD^positive^ group (red). (B) Volcano plot of the unadjusted differential expression analysis. Dashed lines mark the significance thresholds. (C) GSEA of diagnostic RNA sequencing data demonstrating significant enrichment of hallmark pathways in the *FLT3*‐ITD^positive^ group (red) compared with the *FLT3*‐ITD^lost^ group (blue). (D–E) Representative GSEA enrichment plots showing a significant diagnostic enrichment of inflammatory and immune‐related hallmark pathways, including the IL6–JAK–STAT3 signaling, inflammatory response, and interferon‐alpha/gamma response pathways, in the *FLT3*‐ITD^positive^ group. (F) Comparison of representative hallmark pathway enrichment scores (ssGSEA) between the *FLT3*‐ITD^lost^ (blue) and the *FLT3*‐ITD^positive^ (red) groups. Boxes represent interquartile ranges with median values; *p* values were calculated using two‐sided statistical tests. (G) Summary dot plot of differentially enriched hallmark pathways between evolution groups, ranked by the median difference in enrichment score. Dot size reflects ‐log10(*p*) values. (H) Deconvolution of immune cell proportions at diagnosis using CIBERSORTx. Significance levels are indicated as follows: *p < 0.05, **p < 0.01. Proportions are computationally inferred and cannot distinguish leukemic monocyte blasts from non‐malignant monocytes.

**Figure:**
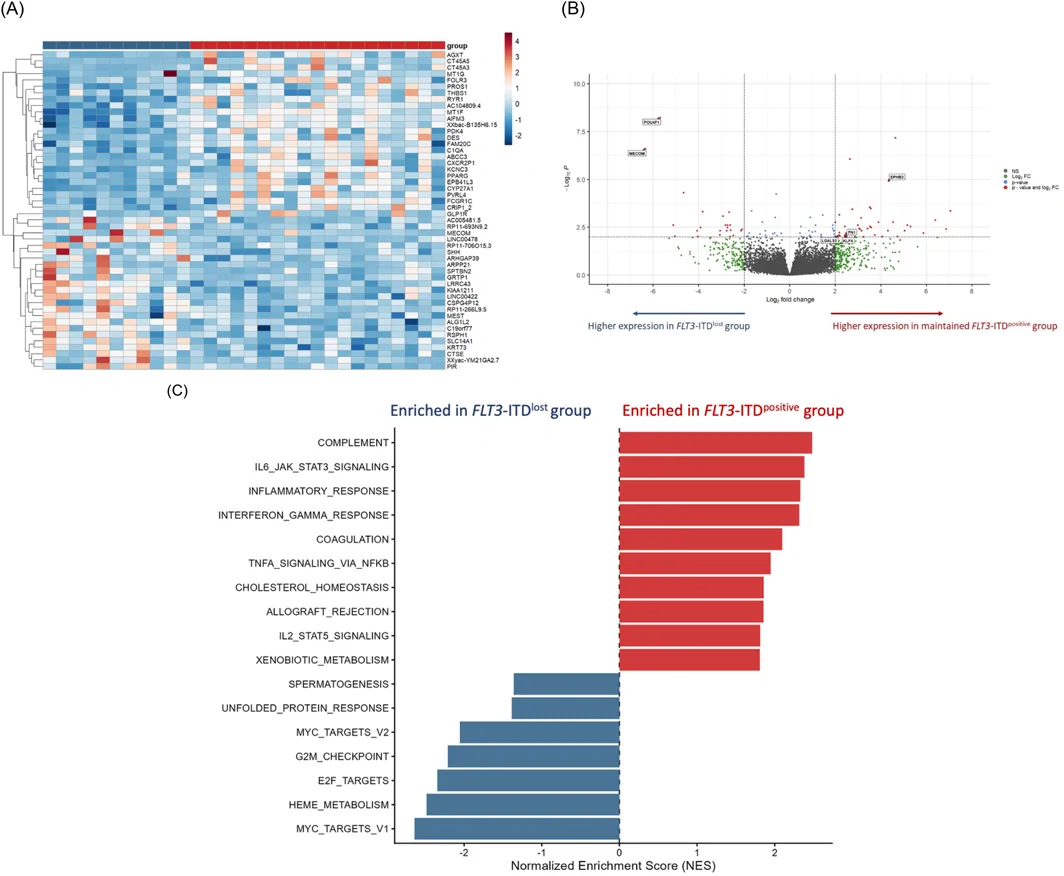

**Figure:**
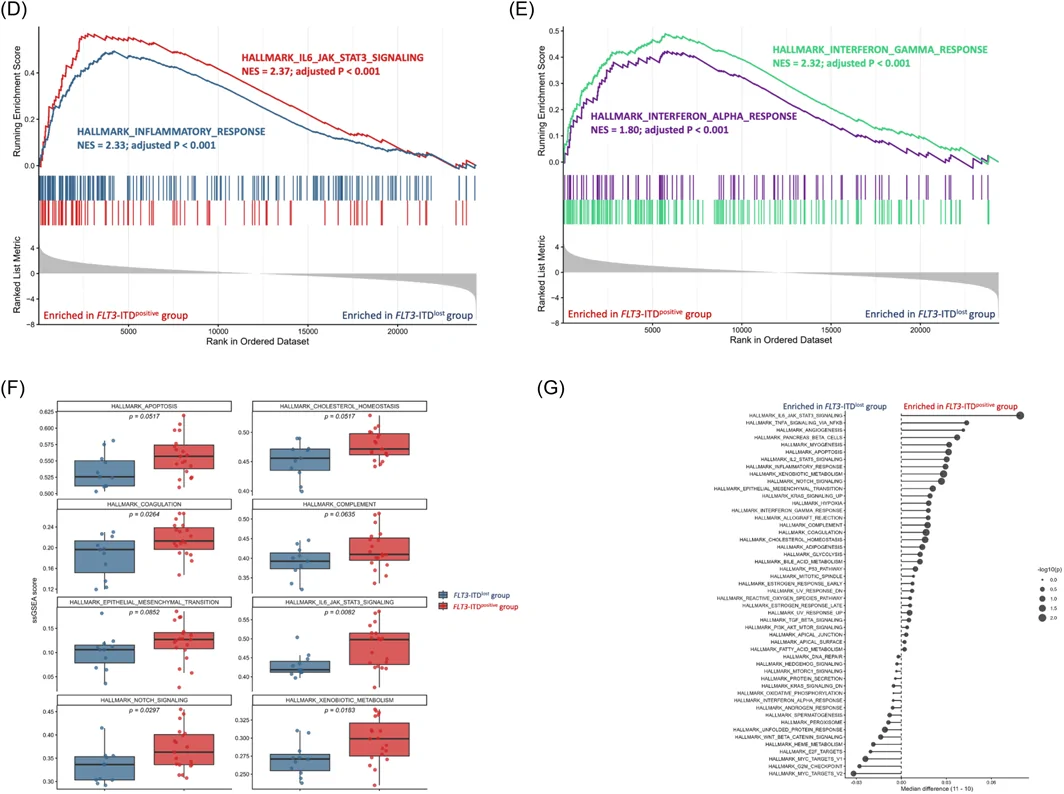

**Figure:**
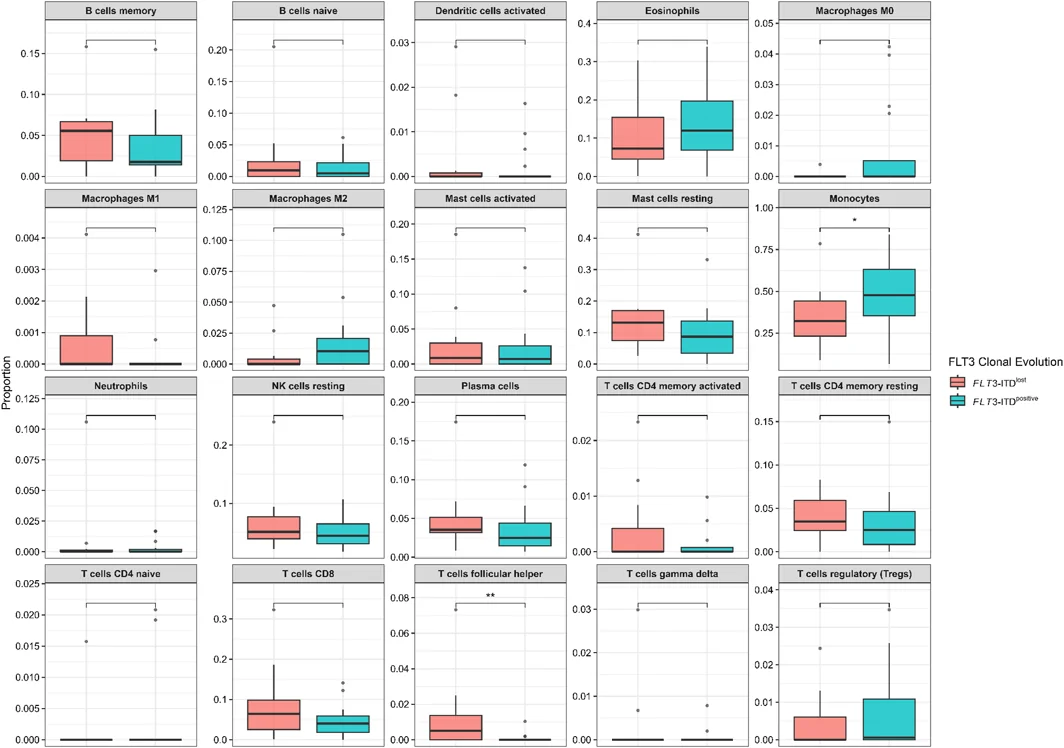

We next performed GSEA to elucidate the functional networks underlying these transcriptomic differences. In unadjusted GSEA, we found that *FLT3*‐ITD^positive^ patients exhibited significant enrichment in inflammation‐related pathways at diagnosis, such as the IL‐6/JAK/STAT signaling pathway, inflammatory response, and interferon‐alpha/gamma pathways (Figure 3C–E). After full adjustment for AR, *NPM1*, and *DNMT3A* mutation status, inflammatory response, interferon‐gamma response, IL‐6/JAK/STAT3 signaling, TNF‐α signaling via NF‐κB, and allograft rejection remained significant (all adjusted *p* < 0.01, Supporting Information S1: Table S10). Sample‐level analysis via single sample gene set enrichment analysis (ssGSEA) showed that, in the unadjusted comparison, patients who maintained *FLT3*‐ITD at relapse exhibited significantly higher enrichment scores for IL‐6/JAK/STAT3 and NOTCH signaling at diagnosis (Figure 3F and 3G). To determine whether these sample‐level signals reflected clone size, we correlated each score with continuous AR. The IL‐6/JAK/STAT3 score correlated significantly with AR (*p =* 0.008), whereas the inflammatory response, interferon‐gamma response, and NOTCH signaling scores did not. In a four‐group DESeq2 likelihood‐ratio analysis, 1423 genes significantly differed across groups (adjusted *p* < 0.05). *MECOM* remained significant in the four‐group LRT (adjusted *p =* 1.6 × 10^−4^) and was consistently downregulated in the *FLT3*‐ITD^positive^ group relative to all three other trajectory groups in pairwise contrasts; inflammatory and IFN‐γ pathway enrichment was confirmed across the four‐group framework (Supporting Information S1: Table S11).

Finally, CIBERSORTx deconvolution showed a greater inferred proportion of monocytes and a lower proportion of T follicular helper cells in patients who maintained *FLT3*‐ITD (Figure 3H). We note that bulk deconvolution cannot distinguish leukemic monocytic blasts from non‐malignant monocytes, and that the *FLT3*‐ITD^positive^ group contained a higher proportion of FAB M4 cases (38.8%); this inferred monocyte enrichment should therefore be interpreted with caution.

## 4 Discussion

This study characterized the clonal dynamics of *FLT3*‐ITD from diagnosis to relapse in AML. By classifying patients into four trajectory groups, we found that the persistence of *FLT3*‐ITD through relapse is strongly associated with the concurrent presence of *NPM1* or *DNMT3A* mutations, and this genomic constellation is associated with poor OS. Second, patients whose *FLT3*‐ITD clones were maintained at relapse already displayed enrichment of inflammatory signaling pathways and an inferred monocyte‐enriched profile at diagnosis; whether this reflects a genuine microenvironmental difference or the morphological composition of the leukemic blasts themselves could not be resolved with bulk data.

The *FLT3*‐ITD^positive^ group was clinically distinct, with markedly higher frequencies of *NPM1* and *DNMT3A* mutations. The co‐occurrence of *FLT3*‐ITD, *NPM1*, and *DNMT3A* mutations constitutes a canonical high‐risk molecular constellation, and their coordinated persistence from diagnosis through relapse indicates strong clonal dominance [25, 26, 27]. This triad was described by Shlush et al., who reported that *DNMT3A*‐mutated cells detected in remission represent preleukemic hematopoietic stem cells that predate *NPM1* and *FLT3*‐ITD acquisition [25], and by Metzeler et al., who reported that *DNMT3A* comutation substantially worsens prognosis in *NPM1*‐mutated AML [27]. Thus, persistence of *DNMT3A* and *NPM1* may provide a stable ancestral framework that facilitates retention of *FLT3*‐ITD through therapeutic selection [26, 28, 29, 30]. Consistent with this model, after excluding *NPM1* and *DNMT3A* from the mutation count, the *FLT3*‐ITD^positive^ group carried significantly fewer additional co‐mutations than the *FLT3*‐ITD^negative^ group, suggesting that this ancestral *DNMT3A*–*NPM1* backbone may reduce the selective pressure for acquisition of additional mutations. Although the *FLT3*‐ITD^positive^ group had a higher AR at diagnosis, AR did not predict trajectory, whereas concurrent *NPM1* and *DNMT3A* mutations did. This suggests that co‐mutation context, rather than clone size, better distinguishes *FLT3*‐ITD trajectories.

In contrast, the *FLT3*‐ITD^lost^ group displayed a predominantly *FLT3*‐wild‐type clonal structure at relapse, with relatively few cooperating mutations across activated signaling, DNA methylation, and cohesin‐related categories at diagnosis. Two clinical pathways contributed to this pattern: approximately half of the patients underwent HSCT in CR1 before relapsing with *FLT3*‐ITD loss, while the remainder relapsed prior to HSCT. The higher rate of HSCT in CR1 in this group (*p* = 0.028) is clinically relevant and raises the possibility that graft‐versus‐leukemia effects contribute to the selective eradication of *FLT3*‐ITD–bearing clones [13, 31]. Supporting this hypothesis, post‐transplant relapses were significantly more likely to show loss of *FLT3*‐ITD than relapses in non‐transplanted patients (*p* = 0.042). However, conditioning‐related selective pressure and baseline differences between transplanted and non‐transplanted patients cannot be excluded; this observation should therefore be considered hypothesis‐generating and warrants validation in further studies. The *FLT3*‐ITD^acquired^ group showed a heterogeneous mutational spectrum without enrichment of *NPM1* or *DNMT3A*, indicating that *de novo FLT3*‐ITD emergence at relapse is not associated with the classic high‐risk triad. These cases most likely reflect the expansion of minor preexisting *FLT3*‐ITD–positive subclones that were below diagnostic detection thresholds and were then selected under therapeutic pressure [15]. Classification as “acquisition” therefore depends on assay sensitivity, as the standard capillary electrophoresis fragment analysis recommended by ELN [18], with a detection limit of approximately 1%–3% variant allele frequency, would classify low‐level subclones as wild type. Consistent with this interpretation, single‐cell studies have demonstrated that *FLT3*‐ITD clones present at variant allele frequencies less than 1% at diagnosis can later expand to clonal dominance at relapse [29, 30], highlighting the potential value of higher‐sensitivity approaches such as next‐generation sequencing or digital PCR.

Among the genes differentially expressed at diagnosis, only *MECOM* remained significantly associated with *FLT3*‐ITD trajectory after adjustment for *NPM1* and *DNMT3A* co‐mutation status and for *FLT3*‐ITD AR. *MECOM* expression was lower in patients who maintained *FLT3*‐ITD at relapse. Because high EVI1 expression from the *MECOM* locus is largely mutually exclusive with *NPM1* mutation [32, 33], this finding is consistent with the marked enrichment of *NPM1* mutations in the *FLT3*‐ITD^positive^ group. However, the association between low *MECOM* expression and *FLT3*‐ITD persistence remained significant after adjustment for *NPM1* and *DNMT3A* status, suggesting that it is not simply a surrogate for this co‐mutation pattern. These findings support an EVI1‐low transcriptional context as an additional feature associated with *FLT3*‐ITD persistence.

Among the inflammatory gene sets enriched at diagnosis in *FLT3*‐ITD^positive^ patients, the inflammatory response and IFN‐γ response signatures were the most robust, remaining significant after adjustment for *FLT3*‐ITD AR and co‐mutation status. Neither signature correlated with *FLT3*‐ITD clone burden. Chronic IFN‐γ exposure can upregulate immune checkpoints such as PD‐L1 and IDO1 on AML blasts and has been correlated with venetoclax resistance [34]. An IFN‐γ‐enriched state at diagnosis may therefore promote leukemic persistence through immune reprogramming rather than tumor clearance. IL‐6/JAK/STAT3 signaling also remained significant after adjustment, although its ssGSEA score correlated with *FLT3*‐ITD AR, indicating partial dependence on clone size. While *FLT3*‐ITD constitutively activates STAT5 [35], supplemental IL‐6‐driven STAT3 activation [36] may create a synergistic antiapoptotic environment [37].

The inferred monocyte enrichment in the *FLT3*‐ITD^positive^ group may have two possible explanations. One is that bone marrow macrophages in AML are skewed toward an immunosuppressive M2‐like polarization [37], providing paracrine cytokine support favoring leukemic survival. Alternatively, the signal may reflect the monocytic phenotype of the leukemic blasts themselves, as monocytic AML blasts transcriptionally resemble mature monocytes. In our cohort, the *FLT3*‐ITD^positive^ group contained a higher proportion of FAB M4 cases (38.8%), and in murine models *NPM1*/*FLT3*‐ITD leukemias likewise show monocytic differentiation [38]. Therefore, whether the observed monocyte enrichment reflects the leukemia microenvironment or blast differentiation cannot be resolved from bulk transcriptomic data and will require single‐cell analysis.

Several limitations warrant consideration. First, our transcriptomic analysis was restricted to diagnostic bulk RNA‐seq in a retrospective cohort, precluding cell‐specific attribution of the observed expression and immune‐cell signatures. Second, although *FLT3*‐ITD was assessed with an analytical sensitivity of 1% [22], minor subclones below this detection threshold may have been misclassified as *FLT3*‐ITD‐negative. Third, the cohort consisted entirely of patients who received intensive chemotherapy. The clonal dynamics of *FLT3*‐ITD under concurrent targeted *FLT3* inhibitors as the front‐line setting or low‐intensity therapy may differ from those described here. While prior studies have characterized clonal evolution under *FLT3* inhibitor therapy [10, 11, 14, 15], integrated transcriptomic analyses remain lacking, which supports the value of further investigation.

In conclusion, *FLT3*‐ITD clonal evolution is associated with the intrinsic architecture present at diagnosis. The persistence of *FLT3*‐ITD was associated with an *NPM1*/*DNMT3A* comutational backbone and, after adjustment for co‐mutation status and *FLT3*‐ITD AR, with a subset of inflammatory signatures. The accompanying monocyte‐enriched profile was inferred from deconvolution and requires single‐cell confirmation. These findings suggest that the susceptibility of *FLT3*‐ITD clones to therapeutic eradication is shaped not only by genetic lesions but also by the preexisting microenvironmental context. Integrative approaches that combine genomic, transcriptomic, and immune profiling may therefore improve risk stratification and inform therapeutic strategies that target both leukemic cells and their supporting niche.

## Author Contributions

Conceptualization, Study Design, and Supervision: Xavier Cheng‐Hong Tsai, Hsin‐An Hou. Data Acquisition: Xavier Cheng‐Hong Tsai, Yu‐Sung Chang, Feng‐Ming Tien, Min‐Yen Lo, Yuan‐Yeh Kuo, Mei‐Hsuan Tseng, Yen‐Ling Peng, Chi‐Yuan Yao, Szu‐Chi Yao, Chien‐Chin Lin, Bor‐Sheng Ko, Ming Yao, Hwei‐Fang Tien, Hsin‐An Hou, Wen‐Chien Chou. Formal Analysis and Interpretation of Data: Xavier Cheng‐Hong Tsai, Yu‐Sung Chang, Yuan‐Yeh Kuo, Mei‐Hsuan Tseng, Yen‐Ling Peng, Szu‐Chi Yao, Hwei‐Fang Tien, Hsin‐An Hou, Wen‐Chien Chou. Writing – Original Draft Preparation and Editing: Xavier Cheng‐Hong Tsai, Hsin‐An Hou. All authors reviewed and approved the final manuscript.

## Funding

This work was partially funded by grants from the National Science and Technology Council (Taiwan) (NSTC 111‐2314‐B‐002‐279‐ and 112‐2314‐B‐002‐116‐MY3), the Ministry of Health and Welfare (Taiwan) (MOHW 111‐TDU‐B‐221‐114001 and MOHW 115‐TDU‐B‐221–114004), and Astellas.

## Conflicts of Interest

Research funding and honorarium from Astellas and Novartis: Xavier Cheng‐Hong Tsai and Hsin‐An Hou. No conflicts of interest: Yu‐Sung Chang, Feng‐Ming Tien, Min‐Yen Lo, Yuan‐Yeh Kuo, Mei‐Hsuan Tseng, Yen‐Ling Peng, Chi‐Yuan Yao, Szu‐Chi Yao, Chien‐Chin Lin, Bor‐Sheng Ko, Ming Yao, Hwei‐Fang Tien

## Supporting information


Supporting Information S1

## Data Availability

The data that support the findings of this study are available on request from the corresponding author. The data are not publicly available due to privacy or ethical restrictions.
