# Supplementary material for: Divergent Clonal Trajectories of FLT3‐ITD in Acute Myeloid Leukemia and Their Clinical and Transcriptomic Associations
Source: Hematol Oncol. 2026 Sep 22;44(5):e70261. doi: 10.1002/hon.70261 (PMC13595497; doi:10.1002/hon.70261)
Supplement: Supplementary file 1 [file HON-44-e70261-s001.pdf]

Divergent Clonal Trajectories of *FLT3*-ITD in Acute Myeloid Leukemia and Their Clinical and Transcriptomic Associations

Supplementary Method

Molecular and bioinformatic analyses

Cytogenetic analysis followed previously established methods[1] and was classified according to the refined Medical Research Council (MRC) criteria.[2] Next-generation sequencing (NGS) was conducted with a TruSight myeloid sequencing panel and HiSeq platform (Illumina, San Diego, CA). Library preparation and sequencing followed the manufacturer's protocols, achieving a median reading depth of 11,000x.[3] The variant analysis algorithm for diagnostic samples, which was previously detailed,[3,4] used a minimum variant allele frequency of 5%. Owing to sequencing sensitivity issues, *CEBPA* mutations[5] and *FLT3*-ITD[6] were analyzed via Sanger sequencing and fragment analysis, respectively. *FLT3*-ITD were assessed at initial diagnosis with a sensitivity of 1%[6] and were re-evaluated at relapse. *FLT3*-ITD clonal evolution was defined as the pattern of acquisition or loss and was assessed from AML diagnosis to evidence of relapsed AML. In patients with archived diagnostic material, ITD lesions were additionally characterized by amplicon-based deep sequencing with getITD.[7]

A total of 132 patients had cryopreserved diagnostic bone marrow samples available for RNA sequencing analysis (**Supplementary Table S1**). Libraries were prepared using the TruSeq Stranded mRNA Library Prep Kit (Illumina, San Diego, CA) and sequenced on an Illumina NovaSeq 6000 platform, generating 150 bp paired-end reads. Raw FASTQ files were processed using CLC Genomics Workbench version 25 (Qiagen Bioinformatics). Adapter and quality trimming were both performed with default settings. Trimmed reads were aligned to the human reference genome (GRCh38/hg38) using the RNA-Seq analysis pipeline with default mapping parameters. To investigate the baseline transcriptomic differences between patients who lost *FLT3*-ITD and those who maintained it at relapse, bulk RNA-sequencing data were analyzed. Differential expression analysis was performed using DESeq2, with significance defined as an absolute fold change > 2 and an adjusted P value < 0.01; the same adjusted P threshold was applied to pathway-level results. To identify the biological pathways that drive *FLT3*-ITD clonal evolution, GSEA was conducted using the clusterProfiler and enrichplot R packages. Genes were ranked on the basis of their differential expression statistics, and enrichment was evaluated against the hallmark gene sets obtained via the msigdbr package. To address potential confounding by co-mutation status and clone burden, the analysis was repeated with *NPM1*/*DNMT3A* mutation status, and *FLT3*-ITD allelic ratio (AR) included as covariates in the design formula, both individually and jointly. GSEA was repeated on the ranked test statistics derived from each adjusted model. Single-sample enrichment scores were computed using GSVA and correlated with continuous *FLT3*-ITD AR by Spearman correlation. The transcriptomes were further analyzed using CIBERSORTx[8] to infer immune cell type composition by digital cytometry.

For *FLT3*-ITD molecular information, archived diagnostic and relapse material was additionally sequenced using an amplicon-based assay covering the *FLT3* juxtamembrane and TKD1 regions, with ITDs called by getITD.[7] The median coverage was 1.1×10^6 reads, and the limit of detection of this assay is 0.0003%.

Supplementary Figure S1.

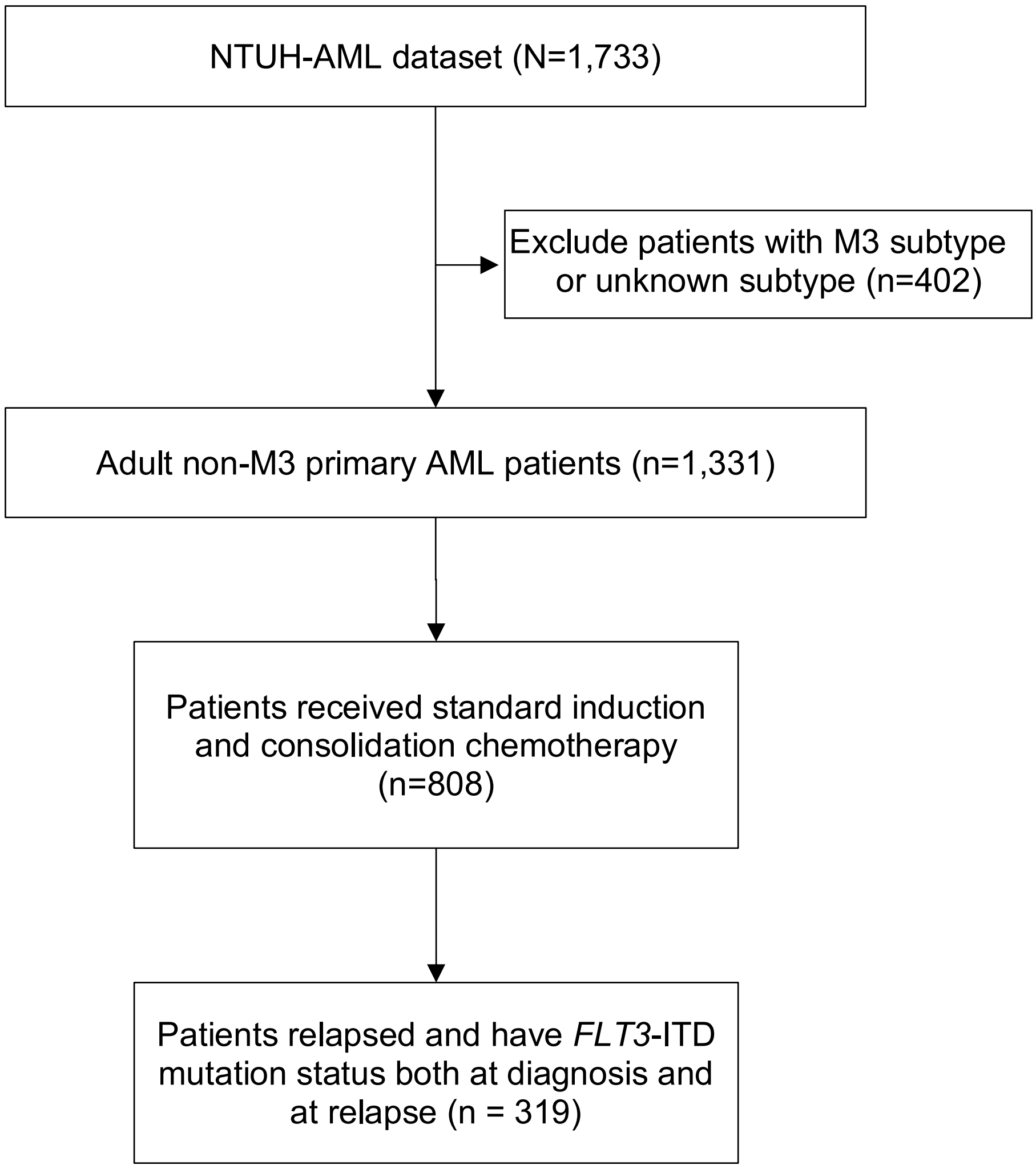

Supplementary Figure S2. Effect of covariate adjustment on the six differentially expressed genes highlighted in the unadjusted analysis. Each panel shows the DESeq2 adjusted P value for one gene across four models. The dashed line marks the significance threshold (adjusted *P*=0.01).

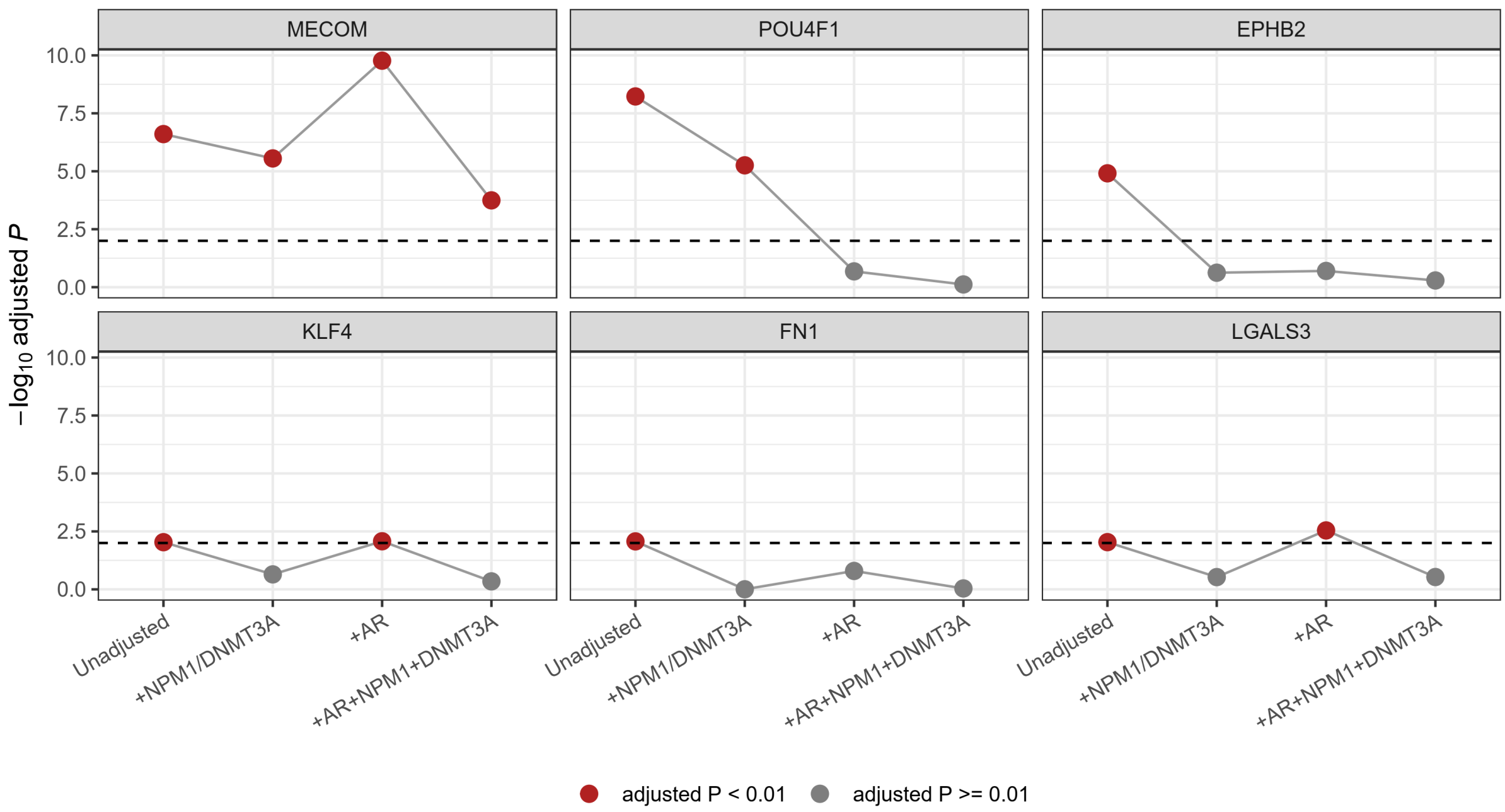

Supplementary Table S1. Comparison between patients with and without RNA sequencing

Variables	Total N = 319	No RNAseq N = 187	RNAseq n = 132	P value
**Sex**$^{\alpha}$				0.212
Male	166 (52.0%)	103 (55.1%)	63 (47.7%)	
Female	153 (48.0%)	84 (44.9%)	69 (52.3%)	
**Age (year)**$^{\beta}$	48.0 (16.1-85.9)	50.3 (16.9-85.9)	46.1 (16.1-81.5)	0.360
**Lab**$^{\beta}$				
WBC (/µL)	20,240 (490-377,130)	15,120 (730-377,130)	25,710 (490-361,300)	0.020
Hb (g/dL)	8.2 (2.7-14.2)	8.2 (3.3-13.5)	8.1 (2.7-14.2)	0.400
Platelet (×1,000 /µL)	49 (3-712)	52 (3-712)	42 (5-396)	0.250
Peripheral blood blast (/µL)	865,920 (0-35,865,063)	736,345 (0-35,865,063)	1,188,985 (0-32,226,750)	0.012
LDH (U/L)	721 (96-7,747)	741 (96-7,293)	695 (140-7,747)	0.525
**FAB**$^{\alpha}$				
M0	5 (1.6%)	4 (2.1%)	1 (0.8%)	0.328
M1	74 (23.2%)	44 (23.5%)	30 (22.7%)	0.867
M2	124 (38.9%)	78 (41.7%)	46 (34.8%)	0.216
M4	94 (29.5%)	52 (27.8%)	42 (31.8%)	0.439
M5	14 (4.4%)	6 (3.2%)	8 (6.1%)	0.221
M6	8 (2.5%)	3 (1.6%)	5 (3.8%)	0.219
**2022 International Consensus Classification**$^{\alpha\gamma}$				
AML with t(8;21)(q22;q22.1)/*RUNX1*::*RUNX1T1*	32 (10.0%)	18 (9.6%)	14 (10.6%)	0.744
AML with inv(16)(p13.1q22) or t(16;16)(p13.1;q22)/*CBFB*::*MYH11*	15 (4.7%)	3 (1.6%)	12 (9.1%)	0.002
AML with t(9;11)(p21.3;q23.3)/*MLLT3*::*KMT2A*	2 (0.6%)	1 (0.5%)	1 (0.8%)	0.804
AML with other *KMT2A* rearrangements	17 (5.3%)	13 (7.0%)	4 (3.0%)	0.125
AML with t(6;9)(p22.3;q34.1)/*DEK*::*NUP214*	2 (0.6%)	0 (0%)	2 (1.5%)	0.091
AML with inv(3)(q21.3q26.2) or t(3;3)(q21.3;q26.2)/*GATA2*; *MECOM*(EVI1)	4 (1.3%)	2 (1.1%)	2 (1.5%)	0.725
AML with other rare recurring translocations	13 (4.1%)	8 (4.3%)	5 (3.8%)	0.827
AML with t(9;22)(q34.1;q11.2)/*BCR*::*ABL1*	2 (0.6%)	2 (1.1%)	0 (0%)	0.233
AML with mutated *NPM1*	71 (22.3%)	48 (25.7%)	23 (17.4%)	0.081
AML with in-frame bZIP *CEBPA* mutations	40 (12.5%)	21 (11.2%)	19 (14.4%)	0.401
AML with mutated *TP53*	6 (1.9%)	4 (2.1%)	2 (1.5%)	0.686

AML with myelodysplasia-related gene mutations	44 (13.8%)	29 (15.5%)	15 (11.4%)	0.290
AML with myelodysplasia-related cytogenetic abnormalities	15 (4.7%)	10 (5.3%)	5 (3.8%)	0.517
AML not otherwise specified (NOS)	56 (17.6%)	28 (15.0%)	28 (21.2%)	0.149
**2022 ELN$^{\alpha}$**				
Favorable	123 (38.6%)	67 (35.8%)	56 (42.4%)	0.233
Intermediate	107 (33.5%)	65 (34.8%)	42 (31.8%)	0.584
Unfavorable	89 (27.9%)	55 (29.4%)	34 (25.8%)	0.474
**HSCT at CR1$^{\alpha}$**				
**NGS MRD available patient number**	127	54	73	
NGS MRD negativity after induction$^{\delta}$	56 (44.1%)	23 (42.6%)	33 (45.2%)	0.769
NGS MRD negativity after 1st consolidation$^{\delta}$	79 (62.2%)	31 (57.4%)	48 (65.8%)	0.338
***FLT3*-ITD clonal fate**				
FLT3-ITD^{negative}	212 (66.5%)	130 (69.5%)	82 (62.1%)	0.168
FLT3-ITD^{acquired}	31 (9.7%)	11 (5.9%)	20 (15.2%)	0.006
FLT3-ITD^{lost}	27 (8.5%)	16 (8.6%)	11 (8.3%)	0.944
FLT3-ITD^{positive}	49 (15.4%)	30 (16.0%)	19 (14.4%)	0.687

Abbreviations: AML, acute myeloid leukemia; FAB, French-American-British classification; Hb, hemoglobin; CR, complete remission; ELN, European LeukemiaNet; LDH, lactate dehydrogenase; WBC, white blood cell; MRD, measurable/minimal residual disease; WHO, World Health Organization; NOS, not otherwise specified;

$^{\alpha}$number of patients (%)

$^{\beta}$median (range)

$^{\gamma}$No patients in the group of AML with other *MECOM* rearrangement

Supplementary Table S2. ICC categories of patients with different trajectories

**Variables**	***FLT3*-ITD[negative] (n = 212, 66.5%)**	***FLT3*-ITD[acquired] (n = 31, 9.7%)**	***FLT3*-ITD[lost] (n = 27, 8.5%)**	***FLT3*-ITD[positive] (n = 49, 15.4%)**	***P* value**
**2022 International Consensus Classification[ab]**					
AML with t(8;21)(q22;q22.1)/*RUNX1*::*RUNX1T1*	24 (11.3%)	4 (12.9%)	2 (7.4%)	2 (4.1%)	0.423
AML with inv(16)(p13.1q22) or t(16;16)(p13.1;q22)/*CBFB*::*MYH11*	14 (6.6%)	1 (3.2%)	0 (0%)	0 (0%)	0.132
AML with t(9;11)(p21.3;q23.3)/*MLLT3*::*KMT2A*	1 (0.5%)	1 (3.2%)	0 (0%)	0 (0%)	0.270
AML with other *KMT2A* rearrangements	15 (7.1%)	0 (0%)	2 (7.4%)	0 (0%)	0.111
AML with t(6;9)(p22.3;q34.1)/*DEK*::*NUP214*	0 (0%)	0 (0%)	0 (0%)	2 (4.1%)	0.056
AML with inv(3)(q21.3q26.2) or t(3;3)(q21.3;q26.2)/*GATA2*; *MECOM*(EVI1)	4 (1.9%)	0 (0%)	0 (0%)	0 (0%)	>0.999
AML with other rare recurring translocations	10 (4.7%)	2 (6.5%)	1 (3.7%)	0 (0%)	0.430
AML with t(9;22)(q34.1;q11.2)/*BCR*::*ABL1*	2 (0.9%)	0 (0%)	0 (0%)	0 (0%)	>0.999
AML with mutated *NPM1*	28 (13.2%)	6 (19.4%)	7 (25.9%)	30 (61.2%)	<0.001
AML with in-frame bZIP *CEBPA* mutations	28 (13.2%)	1 (3.2%)	2 (7.4%)	1 (2.0%)	0.049
AML with mutated *TP53*	8 (3.8%)	0 (0%)	0 (0%)	0 (0%)	0.461
AML with myelodysplasia- related gene mutations	34 (16.0%)	4 (12.9%)	5 (18.5%)	4 (8.2%)	0.501
AML with myelodysplasia-related cytogenetic abnormalities	7 (3.3%)	2 (6.5%)	0 (0%)	4 (8.2%)	0.260
AML not otherwise specified	37 (17.5%)	10 (32.3%)	8 (29.6%)	6 (12.2%)	0.064

Abbreviations: AML, acute myeloid leukemia; ICC, International Consensus Classification
[a]number of patients (%)
[b]No patients in the group of AML with other *MECOM* rearrangement

Supplementary Table S3. Comparison of relapse timing and *FLT3*-ITD trajectories

	FLT3-ITD^{lost} at relapse	*FLT3*-ITD^{positive} at relapse
Post-HSCT relapse (n=17)	10 (58.8%)	7 (41.2%)
Non-HSCT relapse (n=59)	17 (28.8%)	42 (71.2%)

Fisher's exact *P*=0.042; OR=3.46 (95%CI 1.01–13.3)

Supplementary Table S4. (A) Fate of co-mutations from diagnosis to relapse, by functional category (B) Co-mutation status at diagnosis and relapse in patients with *FLT3*-ITD acquisition or loss

(A)

Functional category	Mutations at diagnosis	Retained at relapse	Emerged at relapse
DNA methylation	11	10 (91%)	0
NPM1 mutation	6	4 (67%)	0
MDS-related	11	6 (55%)	2
Activated signaling	10	3 (30%)	1
Tumor suppressor	4	2 (50%)	1
Transcription	3	2 (67%)	1
**Total**	**45**	**27 (60%)**	**5**

(B)

UPN	Trajectory	Mutations at diagnosis	Mutations at relapse	Retained	Lost	Emerged
133	*FLT3*-ITD^{acquired}	*KIT*	*KIT*	*KIT*	—	—
134	*FLT3*-ITD^{acquired}	*NPM1, NRAS, TP53*	*NPM1, TP53*	*NPM1, TP53*	*NRAS*	—
135	*FLT3*-ITD^{acquired}	*KIT*	—	—	*KIT*	—
141	*FLT3*-ITD^{acquired}	*NPM1, TET2*	*NPM1, TET2*	*NPM1, TET2*	—	—
144	*FLT3*-ITD^{acquired}	*DNMT3A, IDH2*	*DNMT3A, IDH2*	*DNMT3A, IDH2*	—	—
157	*FLT3*-ITD^{acquired}	*NRAS*	*NRAS*	*NRAS*	—	—
170	*FLT3*-ITD^{acquired}	*BCOR*	—	—	*BCOR*	—
177	*FLT3*-ITD^{acquired}	*IDH1*	*IDH1*	*IDH1*	—	—
196	*FLT3*-ITD^{acquired}	*STAG2*	—	—	*STAG2*	—
225	*FLT3*-ITD^{acquired}	*U2AF1, WT1*	—	—	*U2AF1, WT1*	—
284	*FLT3*-ITD^{acquired}	*BCOR, RUNX1, STAG2*	*BCOR, RUNX1, STAG2*	*BCOR, RUNX1, STAG2*	—	—
294	*FLT3*-ITD^{acquired}	—	*SRSF2*	—	—	*SRSF2*
314	*FLT3*-ITD^{acquired}	*ASXL1, BCOR, PTPN11, RUNX1*	*ASXL1, BCOR, PTPN11, RUNX1, SRSF2*	*ASXL1, BCOR, PTPN11, RUNX1*	—	*SRSF2*
28	*FLT3*-ITD^{lost}	*BCOR, IDH2, NPM1*	*IDH2, NPM1*	*IDH2, NPM1*	*BCOR*	—
38	*FLT3*-ITD^{lost}	*DNMT3A, NPM1*	*DNMT3A*	*DNMT3A*	*NPM1*	—
39	*FLT3*-ITD^{lost}	*IDH2, NPM1*	—	—	*IDH2, NPM1*	—
140	*FLT3*-ITD^{lost}	*CEBPA, DNMT3A, IDH2, KIT*	*CEBPA, DNMT3A, IDH2*	*CEBPA, DNMT3A, IDH2*	*KIT*	—
194	*FLT3*-ITD^{lost}	*PTPN11*	*GATA2*	—	*PTPN11*	*GATA2*
198	*FLT3*-ITD^{lost}	*NRAS, STAG2*	—	—	*NRAS, STAG2*	—
214	*FLT3*-ITD^{lost}	—	*PTPN11, WT1*	—	—	*PTPN11, WT1*

228	*FLT3*-ITD^{lost}	*WT1*	*WT1*	*WT1*	—	—
267	*FLT3*-ITD^{lost}	*IDH2*, *NRAS*	*IDH2*	*IDH2*	*NRAS*	—
286	*FLT3*-ITD^{lost}	*CEBPA*, *NPM1*, *TET2*	*CEBPA*, *NPM1*, *TET2*	*CEBPA*, *NPM1*, *TET2*	—	—
313	*FLT3*-ITD^{lost}	*CEBPA*, *NRAS*, *WT1*	—	—	*CEBPA*, *NRAS*, *WT1*	—

Supplementary Table S5. *FLT3*-ITD lesion architecture at diagnosis and clonal persistence at relapse

(A) ITD characteristics at diagnosis

Characteristic	*FLT3*-ITD^{lost} (n=25)	*FLT3*-ITD^{positive} (n=46)	P
More than one ITD	21 (84.0%)	35 (76.1%)	N.S.
ITD lesions per patient, median (IQR)	3 (2–4)	3 (2–4)	N.S.
Dominant ITD length, bp, median (IQR)	39 (27–52)	30 (24–48)	N.S.
Insertion site — juxtamembrane domain	16 (64.0%)	32 (69.6%)	N.S.
Insertion site — JMD hinge region	2 (8.0%)	6 (13.0%)	N.S.
Insertion site — TKD1 β-sheet	2 (8.0%)	5 (10.9%)	N.S.
Insertion site — intron 14	5 (20.0%)	3 (6.5%)	N.S.

Abbreviation: N.S., not significant

(B) Persistence of individual diagnostic ITD by insertion site

Insertion site	Lesions at diagnosis	Detected at relapse
Juxtamembrane domain	71	32 (45.1%)
JMD hinge region	20	5 (25.0%)
TKD1 β-sheet	15	4 (26.7%)
Intron 14	12	6 (50.0%)
Total	118	47 (39.8%)

(C) Determinants of ITD persistence

Variable	Odds ratio	95% CI	P
Variant allele frequency at diagnosis, per ten-fold increase	5.00	2.78–9.01	<0.001
ITD length, per base pair	1.00	0.98–1.02	N.S.
Insertion site<br>JMD hinge region versus juxtamembrane domain	0.41	0.14–1.25	N.S.
Insertion site<br>TKD1 β-sheet versus juxtamembrane domain	0.72	0.23–2.25	N.S.
Insertion site<br>intron 14 versus juxtamembrane domain	2.29	0.61–8.66	N.S.

Supplementary Table S6. Multivariate Cox regression adjusting for the 2022 ELN framework with its *FLT3*-ITD–defined criteria removed

Variable	RFS HR (95% CI)	P	OS HR (95% CI)	P
Age[a]	1.005 (0.998–1.012)	0.177	1.014 (1.006–1.023)	0.001
Intermediate vs. Favorable[b]	0.955 (0.714–1.277)	0.757	1.472 (1.060–2.043)	0.021
Adverse vs. Favorable[b]	1.306 (1.003–1.701)	0.047	1.632 (1.210–2.202)	0.001
FLT3-ITD^{lost} vs. *FLT3*-ITD^{negative}	1.167 (0.769–1.771)	0.467	1.005 (0.616–1.640)	0.984
FLT3-ITD^{acquired} or *FLT3*-ITD^{positive} vs. *FLT3*-ITD^{negative}	**1.493 (1.147–1.944)**	**0.003**	**1.457 (1.086–1.954)**	**0.012**
HSCT at CR1[c]	0.985 (0.697–1.392)	0.931	1.177 (0.790-1.754)	0.424

Abbreviations: CI, confidence interval; CR, complete remission; ELN, European LeukemiaNet; HR, hazard ratio; HSCT, hematopoietic stem cell transplantation; OS, overall survival; RFS, relapse-free survival.
[a]Continuous variable.
[b]2022 ELN risk group assigned without the *FLT3*-ITD criteria.
[c]HSCT at CR1 vs. HSCT at other disease status or no HSCT, modeled as a time-dependent covariate

Supplementary Table S7. Multivariate Cox regression adjusting for the 2017 ELN framework with its *FLT3*-ITD–defined criteria removed

Variable	RFS			OS		
	HR	95% CI	*P*	HR	95% CI	*P*
Age[a]	1.006	0.999–1.013	0.102	1.015	1.007–1.024	0.001
Intermediate vs Favorable[b]	1.007	0.764–1.326	0.962	1.432	1.046–1.959	0.025
Adverse vs Favorable[b]	1.394	1.059–1.833	0.018	1.780	1.305–2.427	<0.001
FLT3-ITD^{lost} vs. *FLT3*-ITD^{negative}	1.053	0.698–1.590	0.805	0.968	0.597–1.568	0.893
FLT3-ITD^{acquired} or *FLT3*-ITD^{positive} vs. *FLT3*-ITD^{negative}	1.412	1.084–1.839	0.011	1.410	1.051–1.892	0.022
HSCT at CR1[c]	0.958	0.678-1.354	0.808	1.145	0.769-1.705	0.506

Abbreviations: CI, confidence interval; CR, complete remission; ELN, European LeukemiaNet; HR, hazard ratio; HSCT, hematopoietic stem cell transplantation; OS, overall survival; RFS, relapse-free survival.
[a]Continuous variable.
[b]2017 ELN risk group assigned without the *FLT3*-ITD criteria.
[c]HSCT at CR1 vs. HSCT at other disease status or no HSCT, modeled as a time-dependent covariate

Supplementary Table S8. Survival analysis among patients with *FLT3*-ITD at diagnosis

Variable	RFS			OS		
	HR	95% CI	*P*	HR	95% CI	*P*
**Univariate**						
AR[a]	1.090	1.025–1.160	0.006	1.091	1.028–1.157	0.004
**Multivariable**						
AR[a]	1.084	1.019–1.154	0.011	1.084	1.021–1.151	0.008
Trajectory (positive vs. lost)	1.254	0.758–2.076	0.378	1.219	0.683–2.177	0.503
Age[a]	0.999	0.983–1.016	0.924	1.010	0.991–1.030	0.297
HSCT at CR1[b]	0.631	0.338-1.179	0.149	0.808	0.397-1.643	0.556

Abbreviations: CI, confidence interval; CR, complete remission; HR, hazard ratio; HSCT, hematopoietic stem cell transplantation; OS, overall survival; RFS, relapse-free survival.

[a]Continuous variable.

[b]HSCT at CR1 vs. HSCT at other disease status or no HSCT, modeled as a time-dependent covariate

Supplementary Table S9. Adjusted differential expression analysis of the six genes highlighted in the unadjusted analysis

Gene	log2FC	Unadjusted	+*NPM1*+*DNMT3A*	+AR	+AR+*NPM1*+*DNMT3A*
MECOM	−6.33	2.5×10^{-7}	2.8×10^{-6}	1.7×10^{-10}	1.8×10^{-4}
POU4F1	−5.69	5.9×10^{-9}	5.5×10^{-6}	0.208	0.763
EPHB2	+4.35	1.2×10^{-5}	0.238	0.199	0.513
KLF4	+2.43	0.0092	0.226	0.0084	0.451
FN1	+2.43	0.0085	0.991	0.161	0.912
LGALS3	+2.12	0.0092	0.296	0.0029	0.294

Abbreviations: AR, allelic ratio

Supplementary Table S10. Pathway analysis adjusted for *NPM1*/*DNMT3A* mutation status and *FLT3*-ITD allelic ratio

pathway	NES_unadj	padj_unadj	NES (*NPM1* and *DNMT3A* adjusted)	Padj (*NPM1* and *DNMT3A* adjusted)	NES_AR	padj_AR	NES_Full	padj_Full
HALLMARK_COMPLEMENT	2.477212	6.56E-15	2.286545	4.87E-10	2.448191	2.71E-13	2.341115	3.42E-11
HALLMARK_MYC_TARGETS_V1	-2.63521	8.54E-18	-1.59184	0.001552	-3.00599	1.84E-26	-2.22269	6.83E-10
HALLMARK_TNFA_SIGNALING_VIA_NFKB	1.94408	9.04E-07	1.543944	0.002082	2.247425	6.91E-11	2.185912	6.83E-10
HALLMARK_INFLAMMATORY_RESPONSE	2.335322	6.99E-12	1.923659	2.58E-06	2.296984	2.18E-11	2.041232	7.67E-08
HALLMARK_ALLOGRAFT_REJECTION	1.859237	1.23E-05	1.830057	1.91E-05	1.952523	6.06E-07	1.975427	3.57E-07
HALLMARK_INTERFERON_GAMMA_RESPONSE	2.317351	1.66E-11	2.131003	7.22E-09	2.124306	4.19E-09	1.932719	3.73E-06
HALLMARK_IL6_JAK_STAT3_SIGNALING	2.367505	7.12E-09	2.498171	8.75E-10	2.078863	1.03E-05	1.952926	2.11E-04
HALLMARK_HEME_METABOLISM	-2.47933	1.66E-14	-2.23801	8.75E-10	-1.9912	5.70E-07	-1.72874	3.55E-04
HALLMARK_COAGULATION	2.094038	7.80E-07	1.876633	9.83E-05	2.018204	5.72E-06	1.752936	4.87E-04
HALLMARK_XENOBIOTIC_METABOLISM	1.810724	4.31E-05	1.716537	2.21E-04	1.645619	6.70E-04	1.667544	4.87E-04
HALLMARK_IL2_STAT5_SIGNALING	1.813195	2.83E-05	1.727155	1.21E-04	1.626684	8.10E-04	1.652708	5.96E-04
HALLMARK_MYC_TARGETS_V2	-2.06275	1.73E-04	-1.49075	0.05551	-2.68799	1.30E-10	-1.9943	5.96E-04
HALLMARK_APOPTOSIS	1.785978	1.73E-04	1.792339	1.31E-04	1.923245	6.50E-06	1.716767	5.96E-04
HALLMARK_HYPOXIA	1.495647	0.006539	1.514418	0.004427	1.359768	0.044418	1.573354	0.002213
HALLMARK_G2M_CHECKPOINT	-2.21053	5.00E-10	-1.46574	0.007096	-2.37128	2.29E-12	-1.56044	0.003524
HALLMARK_P53_PATHWAY	1.528282	0.00381	1.561262	0.001552	1.425558	0.014571	1.482801	0.0044
HALLMARK_CHOLESTEROL_HOMEOSTASIS	1.859722	4.52E-04	1.926101	2.76E-04	1.701905	0.003576	1.652733	0.004557
HALLMARK_REACTIVE_OXYGEN_SPECIES_PATHWAY	1.388585	0.076181	1.755438	0.005454	1.4047	0.084805	1.804849	0.008647
HALLMARK_MYOGENESIS	1.784689	4.31E-05	1.812857	3.52E-05	1.443458	0.010152	1.425656	0.010769
HALLMARK_APICAL_JUNCTION	1.473179	0.008011	1.652705	5.51E-04	1.037015	0.469601	1.435218	0.011286
HALLMARK_SPERMATOGENESIS	-1.37251	0.02962	-1.30505	0.103339	-1.38595	0.035508	-1.54748	0.011286
HALLMARK_ADIPOGENESIS	1.541179	0.002517	1.716872	1.69E-04	1.294038	0.045653	1.411377	0.012069
HALLMARK_E2F_TARGETS	-2.34374	1.06E-11	-1.13701	0.238739	-2.55701	3.38E-15	-1.42449	0.01545
HALLMARK_UV_RESPONSE_UP	1.517531	0.00899	1.469861	0.011872	1.46156	0.016792	1.438045	0.01545
HALLMARK_KRAS_SIGNALING_UP	1.713511	2.07E-04	1.539069	0.003389	1.608434	0.001126	1.387096	0.017327
HALLMARK_TGF_BETA_SIGNALING	1.771556	0.004382	1.652816	0.011812	1.487034	0.049505	1.597846	0.017327
HALLMARK_EPITHELIAL_MESENCHYMAL_TRANSITION	1.646735	2.20E-04	1.930043	2.61E-06	1.394143	0.024633	1.340676	0.02213
HALLMARK_HEDGEHOG_SIGNALING	-1.33901	0.125973	-1.10283	0.362616	-1.61787	0.024633	-1.581	0.033213
HALLMARK_NOTCH_SIGNALING	1.736186	0.008011	1.685055	0.011812	1.556557	0.046649	1.467104	0.077539
HALLMARK_UNFOLDED_PROTEIN_RESPONSE	-1.3948	0.041016	-1.04208	0.418879	-1.60052	0.005571	-1.33737	0.083047
HALLMARK_ESTROGEN_RESPONSE_EARLY	1.083967	0.340615	0.993088	0.515123	-1.40961	0.016006	-1.27352	0.097752
HALLMARK_GLYCOLYSIS	1.422272	0.012938	1.679778	3.19E-04	-0.81167	0.94235	1.213507	0.13306
HALLMARK_INTERFERON_ALPHA_RESPONSE	1.796667	2.07E-04	1.77204	3.24E-04	1.462817	0.017589	1.24317	0.138071
HALLMARK_OXIDATIVE_PHOSPHORYLATION	1.383372	0.024301	1.839476	1.36E-05	-1.04121	0.440555	1.171411	0.196308
HALLMARK_ANGIOGENESIS	1.443683	0.076822	1.550431	0.03694	0.929889	0.625632	1.166011	0.30525
HALLMARK_PANCREAS_BETA_CELLS	1.302199	0.153296	1.194486	0.238739	1.189083	0.294286	1.172563	0.30525
HALLMARK_ANDROGEN_RESPONSE	-1.01063	0.473023	-0.80689	0.868852	-0.96479	0.603308	-1.11572	0.330896

HALLMARK_KRAS_SIGNALING_DN	0.960681	0.580271	-0.88546	0.79071	-0.98209	0.601261	-1.09111	0.352364
HALLMARK_BILE_ACID_METABOLISM	1.315941	0.080767	1.169199	0.235578	1.051194	0.448718	1.095182	0.380657
HALLMARK_UV_RESPONSE_DN	1.13362	0.24911	1.010753	0.483092	0.918718	0.678662	-1.06026	0.406879
HALLMARK_PEROXISOME	0.987153	0.50699	1.08198	0.37056	-1.01374	0.511989	-1.05157	0.423096
HALLMARK_MTORC1_SIGNALING	1.054223	0.371174	1.330523	0.022538	-1.28207	0.049505	1.008031	0.499884
HALLMARK_PI3K_AKT_MTOR_SIGNALING	1.447743	0.015759	1.228714	0.152625	1.112252	0.36855	0.984799	0.589821
HALLMARK_ESTROGEN_RESPONSE_LATE	1.142305	0.245545	1.323818	0.03694	-1.20105	0.155574	-0.95116	0.658103
HALLMARK_FATTY_ACID_METABOLISM	1.122209	0.275993	1.283788	0.063277	-0.88199	0.766959	0.925331	0.697599
HALLMARK_MITOTIC_SPINDLE	0.939969	0.629195	1.080343	0.334821	-0.9748	0.601261	0.93351	0.697599
HALLMARK_PROTEIN_SECRETION	1.148107	0.275993	0.867835	0.791023	1.158508	0.304233	0.851118	0.852591
HALLMARK_APICAL_SURFACE	0.854678	0.726079	0.82779	0.799541	0.958728	0.603308	0.778862	0.895929
HALLMARK_WNT_BETA_CATENIN_SIGNALING	1.35869	0.097512	1.186134	0.235578	0.921999	0.64837	-0.7122	0.951413
HALLMARK_DNA_REPAIR	-0.90244	0.726079	1.242564	0.107937	-1.13381	0.276331	0.758403	0.965347

Supplementary Table S11. Four-group simultaneous transcriptomic comparison across *FLT3*-ITD trajectory groups

(A). Pairwise contrasts from joint four-group DESeq2 model for key genes

Gene	LRT adjusted P	pos vs neg log_2FC	pos vs neg adjusted P	pos vs acq log_2FC	pos vs acq adjusted P	pos vs lost log_2FC	pos vs lost adjusted P
MECOM	1.6×10^{-4}	−7.42	1.4×10^{-11}	−4.49	0.009	−6.31	0.009
EPHB2	1.2×10^{-6}	−1.08	0.290	+2.29	0.044	+4.02	0.009
KLF4	0.028	+0.99	0.189	+1.75	0.046	+2.43	0.081
FN1	0.012	−0.88	0.261	+0.14	0.933	+1.78	0.335
POU4F1	0.301	−2.13	0.111	−2.92	0.085	−1.53	0.730
LGALS3	0.112	+0.41	0.637	+0.86	0.377	+2.12	0.104

(B). GSEA on LRT Wald statistic gene ranking (four-group simultaneous)

Pathway	NES	Adjusted P
HALLMARK_INTERFERON_ALPHA_RESPONSE	1.45	0.0004
HALLMARK_INTERFERON_GAMMA_RESPONSE	1.38	0.0002
HALLMARK_ALLOGRAFT_REJECTION	1.35	0.0004
HALLMARK_INFLAMMATORY_RESPONSE	1.34	0.0009
HALLMARK_IL6_JAK_STAT3_SIGNALING	1.33	0.023

Abbreviations: acq, *FLT3*-ITD^{acquired}; LRT, likelihood ratio test; neg, *FLT3*-ITD^{negative}; NES, normalized enrichment score; pos, *FLT3*-ITD^{positive}. log_2FC: positive value = higher expression in *FLT3*-ITD^{positive} vs the comparator group. GSEA gene ranking based on Wald statistic from the LRT-fitted DESeq2 model; positive NES indicates enrichment of the pathway toward genes with higher cross-group variability.

Reference

1. Tsai XC, Hou HA, Tang JL, et al. Genetic alterations and their clinical implications in older patients with acute myeloid leukemia. *Leukemia*. 2016;30(7):1485–1492.
2. Grimwade D, Hills RK, Moorman AV, et al. Refinement of cytogenetic classification in acute myeloid leukemia: determination of prognostic significance of rare recurring chromosomal abnormalities among 5876 younger adult patients treated in the United Kingdom Medical Research Council trials. *Blood*. 2010;116(3):354–365.
3. Tsai CH, Tang JL, Tien FM, et al. Clinical implications of sequential MRD monitoring by NGS at 2 time points after chemotherapy in patients with AML. *Blood Adv*. 2021;5(10):2456–2466.
4. Tsai XC, Hou HA, Tang JL, et al. Prognostic impacts and dynamic changes of cohesin complex gene mutations in de novo acute myeloid leukemia. *Blood Cancer J*. 2017;7(12):663.
5. Lin LI, Chen CY, Lin DT, et al. Characterization of CEBPA mutations in acute myeloid leukemia: most patients with CEBPA mutations have biallelic mutations and show a distinct immunophenotype of the leukemic cells. *Clin Cancer Res*. 2005;11(4):1372–1379.
6. Chou WC, Hou HA, Liu CY, et al. Sensitive measurement of quantity dynamics of *FLT3* internal tandem duplication at early time points provides prognostic information. *Ann Oncol*. 2011;22(3):696–704.
7. Blatte TJ, Schmalbrock LK, Skambraks S, et al. getITD for *FLT3*-ITD-based MRD monitoring in AML. *Leukemia*. 2019;33(10):2535–2539.

8. Newman AM, Steen CB, Liu CL, et al. Determining cell type abundance and expression from bulk tissues with digital cytometry. *Nat Biotechnol*. 2019;37(7):773–782.
